# Pralatrexate in patients with recurrent or refractory peripheral T-cell lymphomas: a multicenter retrospective analysis

**DOI:** 10.1038/s41598-019-56891-0

**Published:** 2019-12-30

**Authors:** Jung Yong Hong, Dok Hyun Yoon, Sang Eun Yoon, Seok Jin Kim, Ho Sup Lee, Hyeon-Seok Eom, Hye Won Lee, Dong-Yeop Shin, Youngil Koh, Sung-Soo Yoon, Jae-Cheol Jo, Jin Seok Kim, Soo-Jeong Kim, Su-Hee Cho, Won-Sik Lee, Jong-Ho Won, Won Seog Kim, Cheolwon Suh

**Affiliations:** 1 Department of Oncology, Asan Medical Center, Ulsan University College of Medicine, Seoul, Republic of Korea; 2 Division of Hematology-Oncology, Department of Medicine, Samsung Medical Center, Sungkyunkwan University School of Medicine, Seoul, Republic of Korea; 3 Department of Internal Medicine, Kosin University Gospel Hospital, Busan, Republic of Korea; 4 Center for Hematologic Malignancies, National Cancer Center, Goyang, Republic of Korea; 5 Department of Internal Medicine, Seoul National University Hospital, Seoul, Republic of Korea; 6 Department of Hematology and Oncology, Ulsan University Hospital, University of Ulsan College of Medicine, Ulsan, Republic of Korea; 7 Department of Internal Medicine, Yonsei University College of Medicine, Seoul, Republic of Korea; 8 Division of Hematology-Oncology, Department of Internal Medicine, Pusan National University Yangsan Hospital, Pusan National University School of Medicine, Yangsan, Republic of Korea; 9 Department of Internal Medicine, Inje University College of Medicine, Inje University Busan Paik Hospital, Gaegumdong, Busanjingu, Busan, Republic of Korea; 10 Division of Hematology & Oncology, Department of Internal Medicine, Soonchunhyang University Seoul Hospital, Seoul, Republic of Korea

**Keywords:** Chemotherapy, T-cell lymphoma

## Abstract

Peripheral T-cell lymphomas (PTCL) are a heterogeneous group of non-Hodgkin’s lymphomas with poor clinical outcomes. Pralatrexate showed efficacy and safety in recurrent or refractory PTCLs. The purpose or this study was to investigate the efficacy and safety of pralatrexate in relapsed or refractory PTCLs in real-world practice. This was an observational, multicenter, retrospective analysis. Between December 2012 and December 2016, a total of 38 patients with relapsed or refractory PTCLs were treated with pralatrexate at 10 tertiary hospitals in Korea. Patients received an intravenous infusion of pralatrexate at a dose of 30 mg/m^2^/week for 6 weeks on a 7-week schedule. Modified dosing and/or scheduling was allowed according to institutional protocols. Median patient age was 58 years (range, 29–80 years) and the most common subtype was peripheral T-cell lymphoma, not otherwise specified (n = 23, 60.5%). The median dosage of pralatrexate per administration was 25.6 mg/m^2^/wk (range, 15.0–33.0 mg/m^2^/wk). In intention-to-treat analysis, 3 patients (7.9%) showed a complete response and 5 patients (13.2%) showed a partial response, resulting in an overall response rate (ORR) of 21.1%. The median duration of response was 7.6 months (range, 1.6–24.3 months). The median progression-free survival (PFS) was 1.8 months (95% confidence interval [CI], 1.7–1.8 months) and the median overall survival was 7.7 months (95% CI, 4.4–9.0 months). The most common grade 3/4 adverse events were thrombocytopenia (n = 13, 34.2%), neutropenia (n = 7, 23.7%), and anemia (n = 7, 18.4%). Our study showed relatively lower ORR and shorter PFS in patients with recurrent or refractory PTCLs treated with pralatrexate in real-world practice. The toxicity profile was acceptable and manageable. We also observed significantly lower dose intensity of pralatrexate in real-world practice.

## Introduction

Peripheral T-cell lymphomas (PTCLs) represent 10% to 15% of non-Hodgkin lymphomas and encompass a heterogeneous group of diseases. The treatment approach for PTCL has traditionally been similar to that for diffuse large B cell lymphoma (DLBCL). However, the prognosis for PTCL is poor with conventional cyclophosphamide, doxorubicin, vincristine and prednisone (CHOP) or CHOP-like regimens, with a 5-year overall survival (OS) of 10–30%, with the exception of anaplastic lymphoma kinase (ALK)-positive ALCL, mycosis fungoides, and subcutaneous panniculitis-like T cell lymphoma^1,2^. These poor clinical outcomes emphasize the urgent need for novel treatment options for patients with PTCL, especially those with relapsed or refractory disease, who have limited response to salvage therapy and an extremely poor prognosis^3,4^.

Novel therapeutic options include monoclonal antibodies (brentuximab vedotin, mogamulizumab), histone deacetylase (HDAC) inhibitors (romidepsin, belinostat, chidamide), phosphoinositide 3-kinase (PI3K) inhibitors (duvelisib, copanlisib) and pralatrexate^4–14^. Over the past years, novel therapeutic options have improved clinical outcomes of PTCLs, but there are still unmet needs in patients with relapsed or refractory disease. Pralatrexate is a novel anti-folate that was designed to be efficiently internalized and to have increased intracellular retention with high affinity for the reduced folate carrier. Pralatrexate was the first agent to receive US Food and Drug Administration (FDA) approval for the treatment of relapsed or refractory PTCL. Early clinical reports of pralatrexate in patients with relapsed or refractory B- or T-cell non-Hodgkin lymphoma showed the tolerability and efficacy of a weekly schedule^7^. In a pivotal multicenter phase 2 study (PROPEL), patients received pralatrexate weekly for 6 weeks of a 7-week cycle, the overall response rate (ORR) was 29% and the median overall survival (OS) was 14.5 months^15^.

Given the rarity and heterogeneity of PTCL, the PROPEL study is the largest data set showing activity of a single agent (pralatrexate), but real world data on the clinical efficacy and safety of pralatrexate are scarce. Based on this background, we performed this multicenter, retrospective analysis to investigate the efficacy and toxicity of pralatrexate in patients with relapsed or refractory PTCLs in real-world practice.

## Results

### Patient characteristics

Table 1 summarizes the baseline clinical characteristics of the 38 patients at the time of pralatrexate initiation. The median age was 58 years (range, 29–80), and the male-to-female ratio was 2.5:1.0. Thirty-two patients (84.2%) had advanced disease (stage III–IV) and 21 patients (55.3%) were in high-intermediate or high international prognostic index (IPI) risk groups. PTCL, not otherwise specified (NOS) (n = 23, 60.5%) was the most common subtype. A majority of patients (n = 21, 55.2%) received pralatrexate as the 4^th^ or greater line of chemotherapy. Only 5 patients (13.2%) received pralatrexate as 2^nd^-line chemotherapy. Eleven patients (28.9%) had relapsed disease after prior autologous (n = 7, 18.4%) or allogeneic (n = 4, 10.5%) hematopoietic stem cell transplantation (ASCT).

**Table 1:** Baseline characteristics.

Variables	N = 38 (%)
**Age** (**years**)	
Median, (range)	58 (29–80)
<65	30 (78.9)
≥65	8 (21.1)
**Sex**	
Male	27 (71.1)
Female	11 (28.9)
**ECOG performance**	
PS 0–1	30 (78.9)
PS 2–4	8 (21.1)
**Extranodal involvement**	
0–1	16 (42.1)
2 or more	22 (57.9)
**LDH elevated**	
No	16 (42.1)
Yes	22 (57.9)
**Stage**	
I – II	6 (15.8)
III - IV	32 (84.2)
**IPI risk group**	
Low/low intermediate	17 (44.7)
High-intermediate/high	21 (55.3)
**PIT risk group**	
Group 1 and 2	25 (65.8)
Group 3 and 4	13 (34.2)
**Pathology**	
PTCL.NOS	23 (60.5)
EATL	4 (10.5)
NKTCL	4 (10.5)
AITL	3 (7.9)
ALCL_ALK negative	2 (5.3)
Transformed MF	2 (5.3)
**Previous chemotherapy lines**	
1	5 (13.2)
2	12 (31.6)
3 or more	21 (55.2)
**Previous HSCT**	
No	27 (71.1)
Autologous	7 (18.4)
Allogeneic	4 (10.5)

Acronyms: ECOG, Eastern Cooperative Oncology Group; LDH, lactic dehydrogenase; IPI, international prognostic index; PIT, prognostic index for PTCL; PTCL NOS, peripheral T-cell lymphoma not otherwise specified; EATL, enteropathy-type T-cell lymphoma; NKTCL, NK-/T-cell lymphoma; nasal type; AITL, angioimmunoblastic T-cell lymphoma; ALCL, anaplastic large-cell lymphoma; ALK, anaplastic lymphoma kinase; MF, mycosis fungoides; HSCT, hematopoietic stem cell transplantation.

Table 2 summarizes the patient distribution by pralatrexate therapy. A total of 43 cycles of pralatrexate was administered in 38 patients with a median of 1.0 cycle per patient (range 1–3 cycles). A total of 149 doses of pralatrexate was administered in all patients with a median of 2.5 doses per patient (range, 1–17 doses). Pralatrexate was administered at lower than the standard dose (30 mg/m^2^/wk). The median dosage of pralatrexate per administration was 25.6 mg/m^2^/wk (range, 15.0–33.0 mg/m^2^/wk). Our analysis allowed for 1week of rest between the 3^rd^ and 4^th^ doses of pralatrexate at the physician’s discretion. No patient finished a complete cycle at the standard dose.

**Table 2:** Administration of pralatrexate.

**Cycles**, **number**	
1 cycle (%)	34 (89.4)
2 cycles (%)	3 (7.9)
3 cycles (%)	1 (2.6)
Total number of cycles, all patients	43
Median number of cycles, (range)	1 (1–3)
**Doses**, **number**	
Total number of doses, all patients	149
Median number of doses per patient, (range)	2.5 (1–17)
**Dosage**, **mg/m**^2^	
Cumulative dose, all patients	3626.0
Mean cumulative dosage per patient	95.4
Median cumulative dosage per patient, (range)	60.0 (15–318)
Mean dosage per administration	25.0
Median dosage per administration, (range)	25.6 (15–30)
Relative dose intensity (%)	35.1

### Efficacy

**Figure 1:**
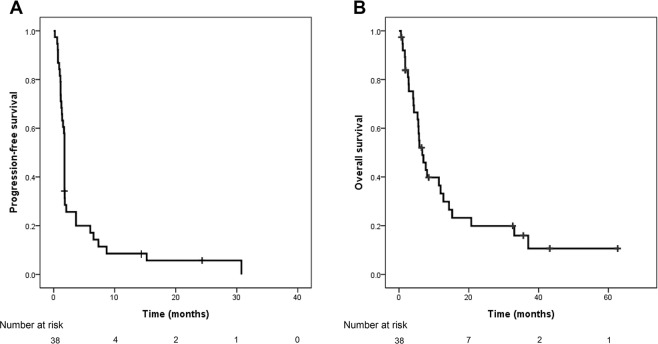
Patient survival. (**A**) Progression-free survival; (**B**) overall survival.

**Figure 2:**
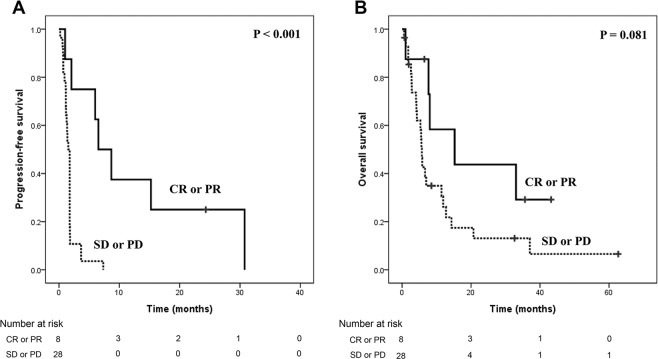
The impact of response to pralatrexate on (**A**) progression-free survival and (**B**) overall survival.

The response and survival outcomes are summarized in Table 3. Of the 38 patients in the study, 3 patients (7.9%) showed a complete response (CR) and 5 (13.2%) showed a partial response (PR), resulting in an ORR of 21.1%. The median duration of response was 7.6 months (range 1.6–24.3 months). The median PFS was 1.8 months (95% confidence interval [CI], 1.7–1.8 months) and the median OS was 6.7 months (95% CI, 4.4–9.0 months). The 1-year expected survival rate was 26.3% (Fig. 1). The PFS and OS did not differ significantly based on the number of chemotherapy regimens administered before pralatrexate. Analysis of PFS and OS based on response (CR or PR) showed that PFS was 6.5 and 1.5 months (*p* < 0.001) and OS was 15.2 and 5.7 months (*p* = 0.081) in responders and non-responders, respectively. (Fig. 2)

**Table 3:** Response and survival outcomes.

Measure	No. of pts (%)
Best response	
Complete response	3 (7.9)
Partial response	5 (13.2)
Stable disease	5 (13.2)
Progressive disease	23 (60.5)
Not available	2 (5.3)
Objective response	8 (21.1)
Median duration of response (range)	7.6 (1.6–24.3)
Survival outcome	Months (95% CI)
Median PFS	1.8 (1.7–1.8)
Median OS	6.7 (4.4–9.0)
1-year expected PFS rate	7.9%
1-year expected OS rate	26.3%

### Toxicity

Treatment-related adverse events are summarized in Table 4. Common grade 3/4 hematological adverse events were thrombocytopenia (n = 13; 34.2%), neutropenia (n = 9; 23.7%), and anemia (n = 7; 18.4%). Febrile neutropenia was observed in 2 patients (5.3%). Grade 3/4 non-hematological adverse events included mucositis (n = 5; 13.1%), pneumonia (n = 1; 2.6%), fatigue (n = 1; 2.6%), and hyperbilirubinemia (n = 1; 2.6%). All grades of mucositis were observed in 16 patients (42.0%). There were 6 patients (15.8%) with grade 1/2 skin rash and 3 patients (7.9%) with grade 1/2 pruritus. There were no treatment-related deaths.

**Table 4:** Grade 3/4 toxicity profiles.

Toxicity profiles (Grade3/4)	No. of pts (%)
**- Hematologic**, **n (%)**	
Neutropenia	9 (23.7)
Anemia	7 (18.4)
Thrombocytopenia	13 (34.2)
Febrile neutropenia	2 (5.3)
**- Non-hematologic**, **n (%)**	
Mucositis	5 (13.1)
Pneumocystis jiroveci pneumonia	1 (2.6)
Fatigue	1 (2.6)
Hyperbilirubinemia	1 (2.6)

## Discussion

There have been remarkable advances in the understanding and management of PTCLs in the last decade. The first of these was gaining an understanding of the molecular pathogenesis of PTCL and subsequent revision of the PTCL classification in the updated 2016 World Health Organization (WHO) classification^16^. The second is the introduction of novel agents such as monoclonal antibodies, HDAC inhibitors, PI3K inhibitors, and anti-folate pralatrexate for treatment of PTCL. Despite this progress, the clinical outcomes of PTCLs are worse than that for B-cell lymphomas and there are still unmet needs for novel treatments. Single-agent pralatrexate therapy received US FDA approval in 2009 for relapsed or refractory PTCLs based on data from the PROPEL study, which predominantly recruited patients in North America and Europe^15^. However, the PROPEL study does not fully reflect the clinical efficacy and safety of pralatrexate in real-world practice due to the prospective nature of the study and European Medicines Agency refused the marketing authorization of pralatrexate due to the lack of comparator and insufficient clinical data.

This study was a multicenter retrospective analysis of pralatrexate therapy in 38 Korean patients with relapsed or refractory PTCLs. The majority of patients in our study had advanced disease (stage III-IV 84.2%) and received pralatrexate as their ≥4th line of chemotherapy (55%). Pralatrexate therapy had an ORR of 21.1% with a CR of 7.9% and median PFS and OS of 1.8 months and 6.7 months, respectively, in our study. Our real-world data revealed a relatively lower objective response rate and shorter PFS compared with the recent prospective Asian data (Japanese and Chinese) showing a promising response rate (45%, 52%) and longer PFS (4.8 months, 5.0 months) (Table 5)^17,18^. One possible explanation is the relatively low dose intensity of pralatrexate in real-world practice. In Korea, the cost of pralatrexate in relapsed or refractory PTCLs is not reimbursed by the national health insurance system. We found that the median dosage per administration of pralatrexate was 25.6 mg/m^2^ (range, 15–30 mg/m^2^) and no patient received a full cycle of pralatrexate at the standard dosage of 30 mg/m^2^ due to financial difficulties and physician discretion. Of note, 6 of the 8 patients who achieved an objective response did not proceed with further cycles of pralatrexate for similar reasons. Our study failed to show clinical benefit in patients who received pralatrexate in the early course of disease compared with patients who received pralatrexate later. We assume that insufficient dose intensity likely weakened the benefit of pralatrexate in this population.

**Table 5:** Summary of pralatrexate in relapsed or refractory peripheral T-cell lymphoma.

Study	Phase	No. of pts	Median age (range)	Line of pralatrexate (≥4th line)	ORR (%)	CR (%)	PFS (months)	Grade 3/4 hematological toxicities	Grade 3/4 mucositis
PROPEL^15^	Prospective Ph. II	111	58 (21–85)	Median prior CTx 3 (range, 1–13)	29%	11%	3.5	Neutropenia 22% Thrombocytopenia 33% Anemia 18%	22%
Japanese^18^	Prospective Ph. I/II	25	71 (42–83)	52%	45%	32%	5.0	Neutropenia 24% Thrombocytopenia 40% Anemia 20%	20%
Chinese^17^	Prospective Ph. II	71	56 (22–77)	43%	52%	24%	4.8	Neutropenia 18% Thrombocytopenia 20% Anemia 24%	20%
Korean	Retrospective	38	58 (29–80)	55%	21%	8%	1.8	Neutropenia 24% Thrombocytopenia 34% Anemia 18%	13%

In terms of toxicity profiles, grade 3/4 hematologic toxicities were thrombocytopenia (34%), neutropenia (24%), and anemia (18%). This finding is consistent with the PROPEL study, Japanese study, and Chinese study (Table 5). The most common grade 3/4 non-hematologic toxicity was mucositis (13.1%). However, other non-hematologic toxicities were mostly grade 1/2 and manageable with supportive care.

Defining optimal salvage and frontline treatment of PTCLs is still a significant challenge. Several clinical trials regarding the efficacy of pralatrexate are ongoing in salvage and frontline settings. Amengual *et al*. showed that the combination of romidepsin and pralatrexate led to an ORR of 71% in patients with relapsed or refractory PTCLs^19^. Advani *et al*. also reported that cyclophosphamide, etoposide, vincristine and prednisone (CEOP) alternating with pralatrexate led to an ORR of 70% and a 2-year PFS rate of 60% as frontline therapy for PTCLs^20^. Several pralatrexate-based combinations, including CHOP (NCT02594267), pembrolizumab plus decitabine (NCT03240211), and durvalumab (NCT03161223), are being watched with keen interest.

This study have several limitations due to small numbers of patients, heterogeneity of the patient population, absence of uniform treatment protocols among participating centers, and several comfounding factors including financial toxicity of pralatrexate. However, this study is one of the largest independent set of data and reflect the non-trial setting practice of pralatrexate.

In summary, our study showed relatively lower ORR and shorter PFS in patients with relapsed or refractory PTCLs treated with pralatrexate in real-world practice compared with the promising results of prospective studies. In this study, we also observed significantly lower dose intensity of pralatrexate owing to financial problems and physician discretion, and we suggest that financial constraints and reimbursement issues pose a significant challenge to the successful completion of pralatrexate therapy in real-world practice.

## Methods

### Trial design and patients

This study was a multicenter, retrospective study using anonymized information from medical charts of patients treated with pralatrexate for relapsed or refractory PTCLs. Between December 2012 and December 2016, a total of 38 patients with relapsed or refractory PTCLs were treated with pralatrexate at ten tertiary hospitals in Korea. Histologically confirmed PTCLs according to the 2016 revision of the World Health Organization classification of lymphoid neoplasm were included^16^. Histologically confirmed PTCLs with the following subtype criteria were excluded in the analysis: (1) aggressive NK-cell leukemia, (2) T-cell prolymphocytic leukemia, (3) T-cell large granular lymphocytic leukemia, and (4) primary cutaneous CD30+ T-cell lymphoproliferative disorders (lymphomatoid papulosis). Pathology review was based on central review of the local pathology report. If there was a need for further examination, ten unstained slides (thickness: ≥3 µm) were sent to the principal investigator’s institution. The primary objective of this study was to evaluate the ORR of pralatrexate. The secondary objectives were to evaluate progression-free survival (PFS), OS and safety profiles. This study was approved by the institutional review board at each hospital and each of the hospitals that approved the study waived the need for informed consent. This study was conducted in accordance with the Declaration of Helsinki.

### Treatment response and toxicity assessment

Pralatrexate was administered as an intravenous push over 3 to 5 minutes at 30 mg/m^2^/wk for 6 weeks followed by 1 week of rest (7-week cycle), along with vitamin B12 (intramuscularly (IM), administered every 8–10 weeks) and folic acid (orally (PO), 1.2 mg/day). Modified dosing and/or scheduling was allowed according to institutional protocols. Treatment was continued until disease progression, unacceptable toxicity, or the patient’s refusal. Treatment responses were evaluated by computed tomography and/or positron-emission tomography (PET) scanning. Treatment responses were evaluated according to the Lugano Classification or the Cheson Criteria based on the availability of a PET scan^21–23^. Safety was assessed by retrospective chart review for adverse events, clinical laboratory results, vital signs, physical examination findings, and electrocardiograms (ECGs) and recorded on a standardized case report form. Adverse events were evaluated according to the National Cancer Institute Common Terminology Criteria for Adverse Events version 4.0.

### Statistics

Descriptive statistics are presented as proportions and medians. Data are also shown as number (%) for categorical variables. PFS was calculated from the initiation of pralatrexate to the first day of disease progression, relapse, or death from any cause. OS was calculated from initiation of pralatrexate to the last follow-up visit or death from any cause. PFS and OS were censored on the last date of follow-up. Survival curves were estimated by the Kaplan–Meier method and the survival distributions were compared using the log-rank test. Statistical analyses were performed using IBM SPSS (IBM Corp. Armonk, NY, USA) v.19.0.

### Ethics approval and consent to participate

Informed consent was not mandatory due to retrospective nature of this study and this study used only anonymized information from medical charts of patients. All procedures performed in this study was approved by the institutional review board at each participating centers and was conducted in accordance with the Declaration of Helsinki 1964. Each of the hospitals that approved the study also waived the need for informed consent.
